# Preliminary Exploration on the Effects of a Novel Antidepressant Formula Food in a Mouse Model

**DOI:** 10.3390/foods14091640

**Published:** 2025-05-07

**Authors:** Xu Chen, Ruoxi Mao, Yunxia Zou, Wentian Yue, Wenwen Dong, Yali Zhang

**Affiliations:** 1 College of Food Science and Nutritional Engineering, China Agricultural University, Beijing 100107, China; xchen10@umass.edu (X.C.); zouyunxia@cau.edu.cn (Y.Z.); yuewentian@cau.edu.cn (W.Y.); 2021306120229@cau.edu.cn (W.D.); 2 Department of Food Science, University of Massachusetts Amherst, Amherst, MA 01003, USA

**Keywords:** depression, high-fat high-sugar diet, gut–brain axis, inflammation, functional food, tail suspension test, forced swimming test, neuroinflammation, hippocampus

## Abstract

Depression poses increasing public health challenges, and alternative dietary strategies are gaining attention for their potential therapeutic effects. This study evaluates a natural ingredient-based formula powder (FP) for its antidepressant effects in a chronic unpredictable mild stress (CUMS) mouse model under both a standard diet and high-fat high-sugar diet (HFHSD) conditions. Behavioral tests demonstrated that FP significantly reduced immobility time in the forced swimming test and tail suspension test, and improved anxiety-like behavior in the open field test, particularly by increasing the central zone activity in mice fed a standard diet. FP supplementation also mitigated CUMS- and HFHSD-induced organ damage, as indicated by increased small intestine and colon coefficients. At the molecular level, FP downregulated the expression of pro-inflammatory cytokines (TNF-α, IL-1β, IL-6) and upregulated brain-derived neurotrophic factor (BDNF) and tryptophan hydroxylase-2 (TPH2) in the hippocampus and colon. These findings suggest that FP exerts antidepressant-like effects by alleviating neuroinflammation and supporting the gut–brain axis, offering a promising functional food approach for managing depression.

## 1. Introduction

“The Global Burden of Disease Study” published in 2019 [1] shows that depressive disorders account for the first place in the burden of disease among mental disorders, and rank 13th in the burden of disease among all diseases globally. On a national and global scale, an increasing prevalence of depression is observed, with a notable trend toward younger age groups being among the affected individuals [2,3,4].

The standard of living in modern society has improved significantly, and diets have shifted from ensuring satiety to focusing on quality. People now have a wider variety of food choices and a general preference for sweet foods. With the ongoing changes in dietary patterns, the proportion of overweight and obese individuals continues to rise, and there is a noticeable shift toward younger age groups. At present, overweight and obesity have become major public health issues, significantly impacting both the physical and mental health of the population [5]. Studies have shown that obesity is associated with an increased risk of depression [6]. This is because obesity is attributed to systemic inflammation, which can potentially contribute to the development of depression [7]. Modern medical research suggests that the causes of depression are highly complex, and its pathogenesis remains incompletely understood. Recently, studies on the gut–brain axis have provided a potential intrinsic mechanism for exploring depression [8,9]. This is reflected in factors such as gut microbiota dysbiosis, elevated inflammation levels, and abnormal neurotransmitter levels observed in patients with depression. Currently, the clinical treatment of depression mainly consists of psychotherapy, electroconvulsive therapy, and pharmacotherapy. Still, these methods are accompanied by side effects such as nausea, insomnia, fatigue, and sexual dysfunction [10]. Therefore, there is a need to develop mild complementary therapies to enhance the efficacy and acceptance of the treatment. Existing studies have demonstrated that natural nutrients in food are related to depression. For instance, polysaccharides exhibit the advantage of multi-component and multi-target effects in preventing major depressive disorder [11,12]; dietary fiber effectively regulates gut microbiota dysbiosis, thereby alleviating depressive symptoms [13,14]; and insufficient intake of vitamin B12 and vitamin D may lead to elevated homocysteine levels, modulated pro-inflammatory cytokines, which in turn contribute to the development of depression [15,16,17]. Functional foods are defined as foods that provide health benefits beyond basic nutrition, including the prevention and management of chronic disease [18]. Based on this concept, our study aims to develop a functional meal replacement powder with natural ingredients to alleviate depression. On the one hand, the formulation of food materials is optimized from the perspective of nutritional health; on the other hand, ingredients rich in anti-depressive components are carefully selected. Specifically, buckwheat flour, flaxseed powder, black fungus powder, broccoli powder, vitamins A, B1, B2, B6, B12, C, D, and folic acid, elastin peptides, and fish oil were chosen as the core raw materials for this study. The ultimate goal is to design a natural ingredient meal replacement powder formula for alleviating depression while ensuring dietary convenience.

To verify the efficacy of the formulated powder, this study establishes a depression animal model in mice with chronic unpredictable mild stress (CUMS). Behavioral experiments, including the open field test (OFT), tail suspension test (TST), and forced swimming test (FST), were conducted to evaluate depressive-like behaviors in mice. Quantitative PCR was employed to measure the expression levels of inflammation-related, neurotrophic, and neurotransmitter-related factors in mice’s hippocampus and colon tissues. Differences between mice fed the formulated powder and those not fed it were analyzed. The results of this study aim to provide a solid experimental foundation for the use of daily foods in the prevention and treatment of depression.

## 2. Materials and Methods

### 2.1. Experimental Animal

A total of 60 SPF grade C57BL/6N mice (female, 1-month-old) were purchased from Beijing Vital River Laboratory Animal Technology Co., Ltd. (Beijing, China). The animals were housed in Polyacrylic cages under standard conditions (20–25 °C, with appropriate humidity and air and a 12 h light:12 h dark cycle). The mice were kept in separate cages, with 3–4 mice per cage, and they were allowed to obtain water and food freely. The experiments were conducted humanely under the 3R (Replacement—Finding alternatives to animal use in research whenever possible, Reduction—Minimizing the number of animals used in experiments to the lowest possible level, and Refinement—Enhancing animal welfare by improving procedures and minimizing suffering) principles of laboratory animals.

### 2.2. Drugs and Reagents

Common Maintenance Feed, High Fat High Sugar Feed (60% fat content) was supplied by Beijing Huafukang Bio-technology Co., Ltd. (Beijing, China). The formulated powder included the following components according to the mass ratio: 50 g buckwheat flour, 20 g elastin peptides, 15 g flaxseed meal, 1 g DHA algal oil, 70 mg green tea powder, 0.7 g black fungus powder, 13.4 g broccoli powder, 55.3 ug vitamin A, 0.9 mg vitamin B1, 0.9 mg vitamin B2, 0.9 mg vitamin B6, 0.16 ug vitamin B12. 6.67 mg vitamin C, 0.67 ug vitamin D, 26.67 ug folic acid. After determining the composition of the formula powder and the proportion of each component, the required formula feed was handed over to Beijing Huafukang Bio-technology Co., Ltd. (Beijing, China) to generate.

### 2.3. Experimental Design

#### 2.3.1. Grouping and Treatment of Mice

A total of 60 SPF mice were acclimated to standard housing conditions for one week. During this period, they were fed a standard diet (SD). After the acclimation period, the body weight of each mouse was measured. The mice were then ear-tagged for identification and randomly assigned into six groups (10 mice per group) using a random number table. The specific grouping details are shown in Table 1.

The standard protocol of CUMS was slightly modified based on the methods described by Nollet [19]. Seven different stressors were applied over a seven-day cycle: tail suspension, swimming, wet bedding, cage tilting, reversed light–dark cycles, shaking, and restraint. This cycle was repeated for four weeks, with the body weight of the mice measured at the end of each seven days. The procedure for establishing the CUMS is detailed in Table 2.

After 4 weeks of CUMS treatment, mice were fasted for 12 h (water was not restricted). Subsequently, the mice were anesthetized and euthanized via cervical dislocation. Body weights were measured, and the mice were dissected. The entire brain, small intestine, and colon were collected and weighed individually. The samples were placed in 1.5 mL EP tubes and stored at −80 °C. Transportation was carried out using liquid nitrogen.

#### 2.3.2. Behavioral Experiments

Mice were randomly selected for the tests, and the evaluators were blinded to the group assignments during behavioral assessments.

#### Open Field Test

An open field test was conducted before and after the CUMS procedure. Each mouse was placed at the center of the arena, and the software started recording from the moment the mouse first crossed the center position. The duration and proportion of time the mouse spent in the center within six minutes were measured. The data were exported to an Excel sheet for statistical analysis using Prism.

#### Tail Suspension Test

Before initiating the CUMS protocol, conduct a tail suspension test (TST). During the CUMS period, perform TSTs regularly. Suspend the mice by the tail at 1 cm from the distal end, using medical adhesive tape to secure the tail to a stand. The test lasts 6 min, and the immobility time is recorded during the final 4 min. Mouse behavior during the test is video-recorded, and the immobility time is analyzed using KEmaze software (SuperTst Version 3.0.0.0) developed by Nanjing Calvin Technology Co., Ltd. (Nanjing, China).

#### Forced Swimming Test

A forced-swimming test was conducted once before the initiation of the CUMS protocol, and subsequent tests were performed periodically during the CUMS procedure. Each mouse was placed in an open cylindrical container (10 cm in diameter, 30 cm in height) filled with water maintained at 23 ± 1 °C to a depth of 20 cm. The test lasted 6 min, and the immobility time was recorded during the final 4 min. After swimming, the mice were immediately removed, towel-dried, and returned to their cages. Mouse behavior during the test was video-recorded, and the immobility time was analyzed using KEmaze software from Nanjing Calvin Technology Co., Ltd. (Nanjing, China).

#### 2.3.3. Organ Index

The organ index was calculated using Formula (1) [20].

(1) $$Organ\;Index=\frac{weight(organ)}{weight(body)}\times 100\%$$

#### 2.3.4. Quantitative Real-Time PCR

The hippocampal and rectal tissues of mice were collected separately and processed according to the instructions of the RNA extraction kit provided by MeganCo., Ltd. (Guangdong, China). The RNA concentration was measured using an RNA analyzer. The system was prepared according to the instructions of the All-in-one First-Strand cDNA Synthesis MasterMix (including gDNase) to remove genomic DNA contamination, followed by the cDNA first-strand synthesis reaction, as per the manufacturer’s guidelines. Primers for the target gene (Table 3) were synthesized by Sangyo Bioengineering Co., Ltd. (Shanghai, China). Glyceraldehyde-3-phosphate dehydrogenase (GAPDH) was used as the internal reference gene, and gene expression levels were detected using a fluorescence quantitative PCR kit.

### 2.4. Statistical Analysis

Data were analyzed using Excel (Microsoft Co., Ltd., version 2019, Redmond, WA, USA) and GraphPad Prism (GraphPad Software, version 8.0.1, San Diego, CA, USA). Quantitative data were represented by mean ± standard error of the mean (SEM). ANOVA followed by a post hoc Least Significant Difference (LSD) test was used to analyze the significance of differences between multiple groups. A *p*-value of less than 0.05 was considered statistically significant. Cohen’s *d* was used to interpret the magnitude of the FP’s effects.

(2) $$d=\frac{M1-M2}{SDpooled}$$

where M_1_ and M_2_ = the means of the two groups; *SD*pooled = pooled standard deviation.

(3) $${\mathrm{SD}}_{\mathrm{pooled}}=\sqrt{\frac{\left(n1-1\right){SD}_{1}^{2}+\left(n2-1\right){SD}_{2}^{2}}{n1+n2-2}}$$

*SD*_1_, *SD*_2_ = the standard deviations of group 1 and 2; n_1_, n_2_ = sample sizes of group 1 and 2.

## 3. Results

### 3.1. Flow Chart of the Animal Experiment

**Figure 1:**
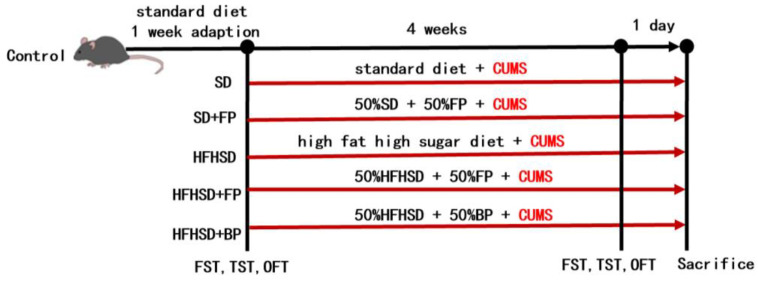
Flow chart of the animal experiment.

The grouping of animals and the time points for behavioral assessments are shown in Figure 1.

### 3.2. The Effect of Formula Powder in Treating CUMS Mice

**Figure 2:**
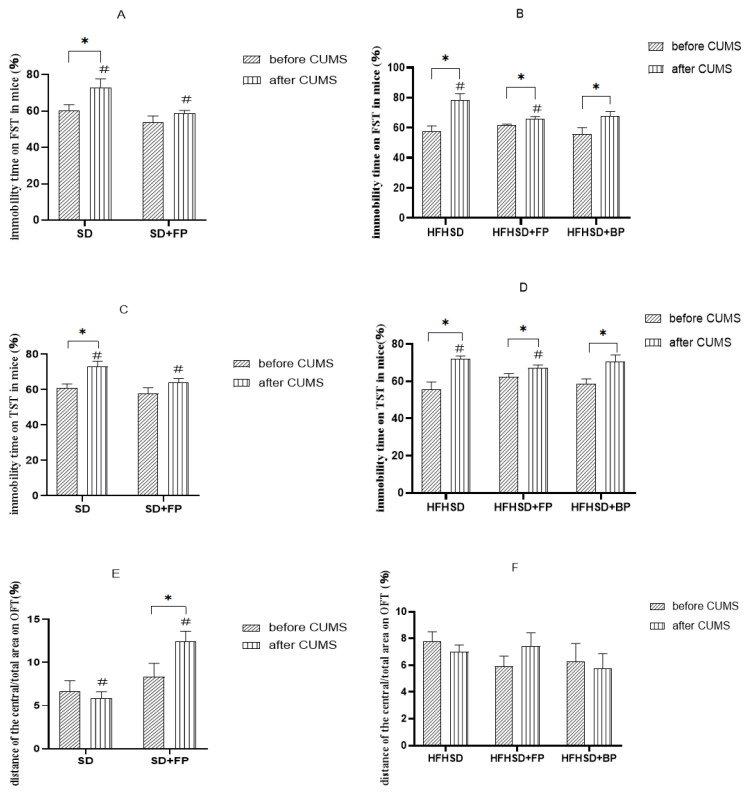
Result of forced swimming test, tail suspension test, and open field test. (**A**) Effect of FP on immobility time in the forced swimming test in mice fed a standard diet (*n* = 10). * Indicates significant difference before and after CUMS (* *p* < 0.05); # indicates significant difference between groups after CUMS (# *p* < 0.05). (**B**) Effect of FP on immobility time in the forced swimming test in mice fed a high-fat high-sugar diet (*n* = 10). * *p* < 0.05 vs. before CUMS; # *p* < 0.05 vs. HFHSD group after CUMS. (**C**) Effect of FP on immobility time in the forced swimming test in mice fed a high-fat high-sugar diet (*n* = 10). * *p* < 0.05 vs. before CUMS; # *p* < 0.05 vs. HFHSD group after CUMS. (**D**) Effect of FP on immobility time in the tail suspension test in mice fed a high-fat high-sugar diet (*n* = 10). * *p* < 0.05 vs. before CUMS; # *p* < 0.05 vs. HFHSD group after CUMS. (**E**) Effect of FP on central/total distance ratio in the open field test in mice fed a standard diet (*n* = 10). * *p* < 0.05 vs. before CUMS; # *p* < 0.05 vs. SD group after CUMS. (**F**) Effect of FP on central/total distance ratio in the open field test in mice fed a high-fat high-sugar diet (*n* = 10). All values are mean ± SEM.

As shown in Figure 2A,B, FP supplementation significantly reduced immobility time in the forced swimming test compared to the non-FP group (*p* < 0.05). Before the CUMS protocol, there were no significant differences in the proportion of immobility time during forced swimming among all groups. After CUMS modeling, the proportion of immobility time during forced swimming was significantly different in all groups, except for the SD+FP group (*p* < 0.05). After CUMS modeling, the proportion of immobility time in the SD+FP group was significantly different from the SD group (*p* < 0.05). Mice on a high-fat high-sugar diet showed similar results. After CUMS modeling, the proportion of immobility time in the HFHSD+FP group was significantly lower than that in the HFHSD group (*p* < 0.05). This suggests that FP can alleviate despair-like behavior and improve the depressive state in mice under different feeding conditions. The proportion of immobility time in the HFHSD+BP group was lower than in the HFHSD group, but there was no significant difference. This indicates that the effect of BP on improving depressive states is not as effective as FP.

According to the results shown in Figure 2C,D, FP had a significant effect on the immobility time during the tail suspension test in mice (*p* < 0.05). Similar to the findings from the forced swimming test, there were no significant differences in the proportion of immobility time among groups before the CUMS protocol. However, after CUMS modeling, all groups, except the SD+FP group, showed statistically significant differences in the proportion of immobility time (*p* < 0.05). Compared to the SD group, the proportion of immobility time in the SD+FP group after CUMS modeling was significantly different (*p* < 0.05). Mice on a high-fat high-sugar diet exhibited similar results: after CUMS modeling, the HFHSD+FP group showed a significantly lower proportion of immobility time compared to the HFHSD group (*p* < 0.05). These results indicate that FP can alleviate despair-like behavior and improve depressive states in mice under different feeding conditions. Although the proportion of immobility time in the HFHSD+BP group was lower than that in the HFHSD group, the difference was not significant, suggesting that BP is less effective than FP in improving depressive states.

Figure 2E,F demonstrates that FP significantly influenced the distance traveled in the open field test’s central area/total area (*p* < 0.05). Before CUMS modeling, no significant differences were observed in the central/total distance ratio among the groups. After CUMS modeling, the central/total distance ratio decreased in all groups except for the SD+FP and HFHSD+FP groups. Compared to the SD group, the SD+FP group exhibited a statistically significant difference in the central/total distance ratio (*p* < 0.05). This suggests that FP can alleviate anxiety-like behavior and improve depressive states in mice on a standard diet. Although the central/total distance ratio increased in the HFHSD+FP group and decreased in the HFHSD+BP group compared to the HFHSD group, neither change reached statistical significance.

In addition, we calculated the effect sizes using Cohen’s *d* to interpret the magnitude of the FP’s effects. In this analysis, M1 refers to the CUMS+SD or CUMS+HFHSD group, and M2 refers to the corresponding FP intervention group (CUMS+SD+FP or CUMS+HFHSD+FP). A positive Cohen’s *d* indicates that FP reduced depression-like behavior (e.g., shorter immobility time or increased exploration), while a negative *d* reflects a shift in the opposite direction.

Most comparisons showed moderate to large effect sizes (*d* > 0.8) (Table 4), suggesting that FP had a meaningful impact on behavioral outcomes. Only the OFT central/total distance ratio between the CUMS+HFHSD group and the CUMS+HFHSD+FP group showed a small and negative effect (*d* = −0.18), which may indicate variability or limited impact in that specific metric.

### 3.3. Effect of Formula Food on Organ Index in CUMS Mice

**Figure 3:**
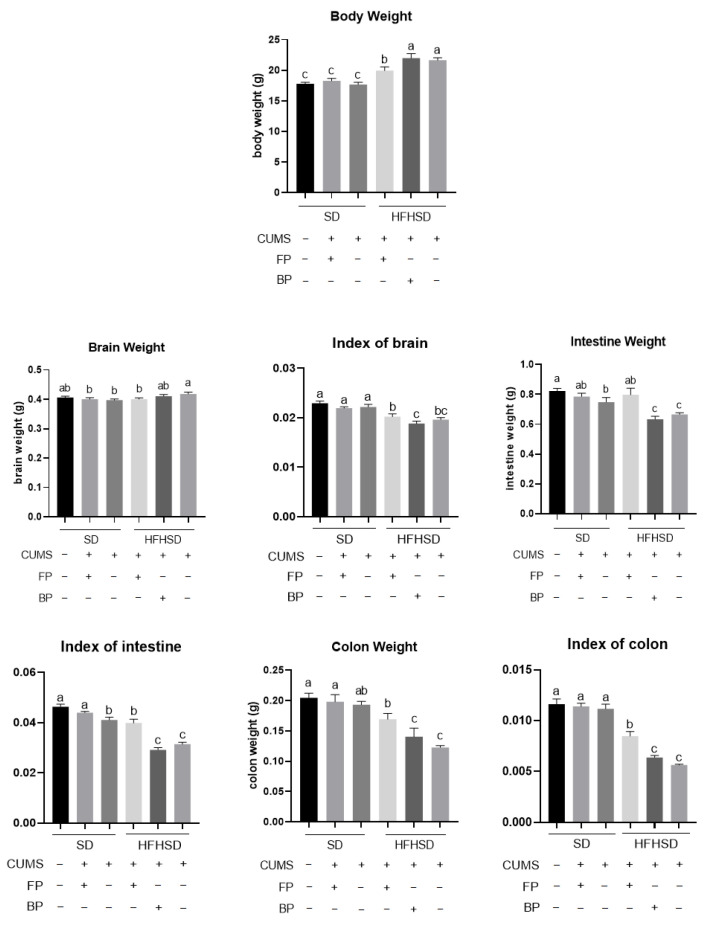
Body weight of mice after 4 weeks of CUMS treatment in each group (*n* = 10). Brain wet weight and brain coefficient of mice in each group [*n* = 10, except control group (*n* = 9) and HFHSD+FP group (*n* = 8)]. Small intestine weight and small intestine coefficient of mice in each group (*n* = 8). Colon weight and colon coefficient of mice in each group [*n* = 10, except HFHSD+FP group (*n* = 8)]. Values are mean ± SEM. Different letters indicate significant differences (*p* < 0.05). Different letters indicate significant differences (*p* < 0.05).

Body weight is a commonly monitored indicator in CUMS models to evaluate the physiological impact of chronic stress and dietary interventions [21]. As shown in Figure 3, mice in the HFHSD group exhibited significantly higher body weights than those in the SD group (*p* < 0.05), indicating that a high-fat high-sugar diet promotes weight gain. Interestingly, the HFHSD+FP group had significantly lower body weights than other high-fat-fed groups, suggesting that FP supplementation may mitigate diet-induced weight gain. This implies that FP may also exert a beneficial effect on weight regulation under obesogenic conditions, potentially through metabolic modulation or anti-inflammatory activity.

Mice in the HFHSD group exhibited significantly higher brain weights than those in the SD+FP, SD, and HFHSD+FP groups (Figure 3), suggesting that excessive fat and sugar intake may affect brain lipid accumulation. To further explore potential changes in brain structure relative to body weight, we examined the brain coefficient, an index often used to evaluate brain atrophy. Brain atrophy can result from multiple factors, including aging, trauma, neurological infections, and diseases such as depression [22]. As shown in Figure 3, FP had no significant effect on the brain coefficient under standard diet conditions. Compared with the control group, the brain coefficients of mice on a high-fat high-sugar diet showed statistically significant differences (*p* < 0.05). The brain coefficient of the HFHSD+FP group was significantly higher than that of the HFHSD+BP group (*p* < 0.05), indicating that the effect of FP was superior to that of BP.

The intestine weight in Figure 3 shows that, compared to the control group, mice in the SD and HFHSD groups had significantly lower small intestine weights (*p* < 0.05), indicating that both CUMS and prolonged high-fat high-sugar diets can induce small intestine atrophy. Figure 3 further demonstrates that the small intestine coefficient in the SD+FP group was significantly higher than that in the SD group, suggesting a protective effect of FP under standard diet conditions. A similar trend was observed under HFHSD conditions, where the HFHSD+FP group exhibited significantly higher small intestine coefficients than both the HFHSD and HFHSD+BP groups (*p* < 0.05). These findings suggest that FP can effectively alleviate small intestine atrophy induced by CUMS and high-fat high-sugar diets and that its efficacy is superior to BP.

The colon weight in Figure 3 shows that mice in the HFHSD group had lower net colon weights compared to those in the other group, demonstrating that long-term HFHSD intake may lead to colonic atrophy. Interestingly, the colon weight of the HFHSD+FP group was significantly greater than that of the HFHSD group, implying that FP supplementation may markedly attenuate HFHSD-induced colonic atrophy. There was no significant effect of FP on the mouse colon coefficient under normal feeding conditions. Compared to the control group, the differences in colon coefficients of mice on high-fat high-sugar diets were all statistically significant (*p* < 0.05). The colon coefficients of mice in the HFHSD+FP group were significantly higher than those in the HFHSD and HFHSD+BP groups. This suggests that FP significantly affects the mouse colonic coefficient in mice under high-sugar high-fat dietary conditions and has a better impact than BP.

### 3.4. Effects of the Formula Food on the Expression of Inflammatory Factors in CUMS Mice

**Figure 4:**
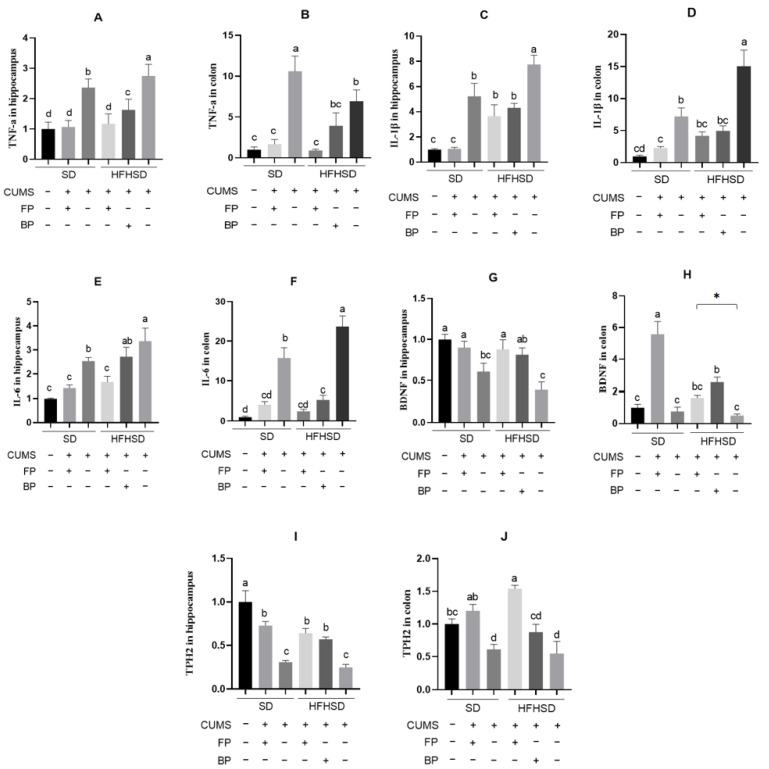
Effect of FP on TNF-α gene expression in the (**A**) hippocampus and (**B**) colon of mice under different diets (*n* = 5). Effect of FP on IL-1β gene expression in the (**C**) hippocampus and (**D**) colon of mice under different diets (*n* = 5). Effect of FP on IL-6 gene expression in the (**E**) hippocampus and (**F**) colon of mice under different diets (*n* = 5). Effect of FP on BDNF gene expression in the (**G**) hippocampus and (**H**) colon of mice under different diets (*n* = 7). * indicates a significant difference between the two groups. Effect of FP on TPH2 gene expression in the (**I**) hippocampus and (**J**) colon of mice under different diets (*n* = 7). All values are mean ± SEM. GAPDH was used as an internal reference. SD group without CUMS was used as a control (set to 1). Different letters indicate significant differences (*p* < 0.05).

Inflammation and adipokines are important mechanisms in the development of depression. Tumor necrosis factor-α (TNF-α), which can affect lipid metabolism and induce inflammatory responses, is a pro-depressive factor [23]. FP significantly affected TNF-α gene expression levels in the hippocampus and colon of mice subjected to different dietary conditions (*p* < 0.05). Following CUMS modeling, TNF-α gene expression levels in the hippocampus and colon were significantly elevated in the SD group (*p* < 0.05). Compared to the SD group, the SD+FP group exhibited statistically significant reductions in TNF-α gene expression levels in both the hippocampus and colon. Similar results were observed under high-fat high-sugar dietary conditions, where the HFHSD+FP group showed significantly lower TNF-α gene expression levels in the hippocampus and colon compared to the HFHSD group. Moreover, as shown in Figure 4A, the TNF-α gene expression levels in the hippocampus of the HFHSD+FP group were significantly lower than those in the HFHSD+BP group. These findings suggest that, compared to buckwheat flour, the intake of formulated powder is more effective in mitigating depressive-like states in mice.

From Figure 4C,D, it can be observed that Interleukin-1 beta, (IL-1β) gene expression levels in both the hippocampus and colon were significantly higher in the SD+CUMS group compared to the SD group without CUMS modeling and the SD+CUMS+FP group (*p* < 0.05). A similar trend was found under high-fat high-sugar feeding conditions, where the IL-1β gene expression levels in the hippocampus and colon of the HFHSD+CUMS group were markedly higher than those in the HFHSD+CUMS+FP group and HFHSD+CUMS+BP group (*p* < 0.05).

Figure 4E,F show that, after CUMS modeling, there was a statistically significant difference in Interleukin-6 (IL-6) levels in the hippocampus and colon between the SD and HFHSD groups (*p* < 0.05), with IL-6 levels in the HFHSD group being significantly higher than those in the SD group (*p* < 0.05). The IL-6 expression levels in the hippocampus and colon of the SD+CUMS and HFHSD+CUMS groups were significantly higher than those in the SD+CUMS+FP and HFHSD+CUMS+FP groups, respectively (*p* < 0.05). Additionally, IL-6 gene expression levels in the hippocampus of the HFHSD+FP group were significantly lower than those in the HFHSD+BP group (Figure 4E). It demonstrates that the intake of formulated powder, compared to buckwheat flour, is more effective in alleviating depressive-like states in mice.

Studies have shown that brain-derived neurotrophic factor (BDNF) levels are negatively correlated with the severity of depression, and BDNF mRNA expressions can serve as a biomarker for the adjunctive diagnosis of depression [24]. The BDNF gene expression levels in the hippocampus of both the SD group and the HFHSD group of mice that underwent CUMS modeling were significantly lower than those in the SD-fed mice that did not undergo CUMS modeling (*p* < 0.05) (Figure 4G). Furthermore, the BDNF gene expression levels in the hippocampus and colon of mice in the HFHSD+FP group were significantly higher than those in the HFHSD group.

Tryptophan hydroxylase 2 (TPH2), which is expressed in both the brain and intestine, is a key rate-limiting enzyme in the synthesis of 5-HT [25,26]. Current research widely believes that the expression of this gene is negatively correlated with the development of depression. Figure 4I,J indicate that, following CUMS modeling, TPH2 gene expression levels in both the hippocampus and small intestine of the SD group mice significantly decreased (*p* < 0.05). Compared to the SD group, the TPH2 gene expression levels in the hippocampus and colon of mice in the SD+FP group showed statistically significant differences. Similar results were observed under high-sugar high-fat feeding conditions. In addition, the TPH2 gene expression levels in the hippocampus and colon of mice in the HFHSD+FP group were significantly higher than those in the HFHSD group.

## 4. Discussion

Depression is a common mental disorder characterized by low mood, reduced interest, pessimism, lack of initiative, poor appetite, and disrupted sleep patterns [27]. Numerous studies have illustrated a correlation between obesity and the onset of depression [28,29,30]. Furthermore, high-fat high-sugar diets are known to contribute to the development of obesity [31]. In our study, mice were fed standard maintenance diets and high-fat high-sugar diets to simulate the complex dietary conditions of humans. The objective was to evaluate the impact of FP supplementation on depression.

**Figure 5:**
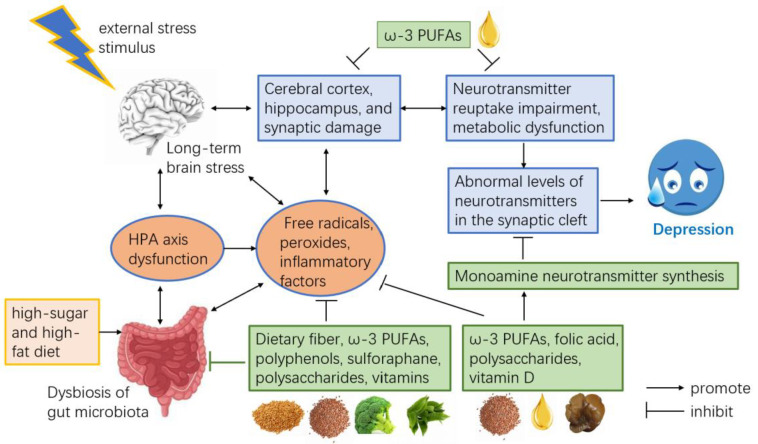
Proposed mechanism of the anti-depressant effects of the functional formula powder.

As a functional meal, our formula mainly contains buckwheat bran powder (a source of high-quality carbohydrates and dietary fiber), elastin peptides, DHA algal oil, flaxseed powder (rich in ω-3 polyunsaturated fatty acids, ω-3 PUFAs), green tea powder (rich in tea polyphenols), black fungus powder (rich in black fungus polysaccharides), broccoli powder (rich in sulforaphane), and a variety of vitamins (including vitamin B6, folate, vitamin D, and vitamin B12). Among them, buckwheat flour and flaxseed flour, both rich in dietary fiber, have been shown to mitigate depressive symptoms through mechanisms such as modulating the transcription of gut-microbiota-related genes, reducing inflammatory cytokine levels, altering intestinal pH and permeability, and enhancing neurotransmitter concentrations [32]. Studies show that low intake of ω-3 PUFAs increases the incorporation of saturated fatty acids (SFAs) and cholesterol into cell membrane phospholipids. This change may restrict the activity of membrane-bound enzymes, reduce receptor number and affinity, and ultimately impair neurotransmitter synthesis and function [33]. Green tea powder is rich in nutrients, particularly polyphenols. Studies have shown that green tea polyphenols can alleviate depressive symptoms in socially defeated depression-model mice [34,35]. Polysaccharides in black fungus powder may ease depressive symptoms by modulating the Trp metabolic pathway and upregulating monoamine neurotransmitters [36]. Therefore, the FP used in this study holds potential antidepressant properties. The proposed mechanism of the anti-depressant effects of the functional formula powder is shown below (Figure 5).

The principle of the open field test is based on the natural aversion of animals to open spaces, coupled with their innate curiosity to explore new environments. Mice with higher levels of anxiety tend to spend a lower proportion of their time and distance in the central area of the open field [37]. In this study, FP alleviated anxiety symptoms in mice on a standard diet, but showed no significant effect on mice fed a high-fat high-sugar diet. This may be attributed to the increased depression threshold induced by the high-fat high-sugar diet and the limited intensity of the CUMS model. The immobility observed in the tail suspension test and forced swim test reflects despair behavior in animals, mimicking depressive states in humans. A higher proportion of immobility time indicates a greater severity of depression in mice [38]. Our study shows that FP significantly reduced the immobility time in both the forced swim test and tail suspension test across different dietary conditions, alleviating depressive symptoms in mice. However, no significant difference was observed in immobility time between the HFHSD+BP group and the HFHSD group, suggesting that the effect of FP was superior to that of its primary component, BP.

In addition, body weight is often used as a supplementary parameter to evaluate stress-induced physiological alterations in animal models of depression as well. In our study, although the SD+CUMS group did not exhibit a significant reduction in body weight compared to the control group, this is not uncommon in CUMS models and may result from variability in stress sensitivity or relatively mild stimulation intensity [19,39]. Nevertheless, the observed increases in immobility time and pro-inflammatory gene expression confirm that the depression-like state was successfully induced. In mice fed a high-fat high-sugar (HFHSD) diet and subjected to CUMS, the brain indices were significantly reduced (*p* < 0.05). However, the absolute brain weight of HFHSD mice was significantly higher than that of the SD group (*p* < 0.05), suggesting that an HFHSD may synergize with CUMS to induce brain atrophy. Since lipids constitute a significant component of the brain [40], a HFHSD might lead to both hypertrophy and hyperplasia of adipocytes within the brain. A key element of the cerebral vascular system is the blood–brain barrier (BBB), which rigorously regulates the exchange of substances between the blood and the brain. This dynamic barrier maintains the stability of the brain microenvironment by preventing neurotoxins from entering and thereby facilitating normal neuronal function [41]. The enlargement and increased number of brain adipocytes may alter neurotrophic factors and oxidative stress levels [42], ultimately compromising the integrity of the BBB. This disruption can result in neuronal damage [43] and reduced synaptic density [44], leading to impaired brain development. Notably, the brain weight of mice in the HFHSD+FP group was significantly lower than that of those in the HFHSD group (*p* < 0.05), indicating that FP supplementation can effectively mitigate the accumulation of adipocytes in the brain. Similarly, intestinal indices were significantly reduced after CUMS induction, suggesting that an HFHSD may also exacerbate pro-inflammatory cytokine levels [45], disrupt gut microbiota homeostasis [46], reduced microbial diversity [47], and led to metabolic dysfunction [48], potentially resulting in brain and intestinal damage. The colon, as a critical component of the gastrointestinal tract, plays an essential role in the absorption of nutrients such as vitamins, water, and inorganic salts, as well as in feces formation. Previous studies have shown a strong correlation between ulcerative colitis and both depression [49] and high-fat high-sugar diets [50]. Nutritional components in FP effectively relieved damage induced by the high-fat high-sugar diet, alleviating the reductions in the whole-brain, small intestine, and colon coefficients.

Chronic stimulation can cause an increase in pro-inflammatory cytokine levels in mice [51]. It aligns with the results of this study, where hippocampal and colonic TNF-α gene expression significantly increased in mice on a standard diet after CUMS modeling. Upregulation of TNF-α activates the NF-κB pathway [52], further stimulating inflammatory responses and inducing the secretion of additional IL-1β and IL-6 cytokines. Experimental results also showed that hippocampal TNF-α, IL-1β, and IL-6 gene expression levels in the HFHSD group were significantly higher than those in the SD group. At the same time, colonic IL-1β and IL-6 gene expression levels were markedly elevated, indicating that a high-fat high-sugar diet increased pro-inflammatory cytokine expression in the hippocampus. Prolonged high-fat high-sugar intake converts excess glucose into triglycerides, which accumulate in adipocytes and cause them to enlarge and proliferate. In response, adipocytes secrete pro-inflammatory cytokines such as TNF-α, IL-1β, and IL-6 in an attempt to reduce lipid content [53]. FP reduced the expression of pro-inflammatory cytokine genes, including TNF-α, IL-1β, and IL-6, in the hippocampus and colon of CUMS model mice. Additionally, it lifted the expression of genes related to BDNF and the serotonin synthesis enzyme TPH2. Compared to BP, FP demonstrated stronger protective effects in mice fed a high-fat high-sugar diet.

## 5. Conclusions

In conclusion, the formulated powder used in this study, whether added to standard or high-fat high-sugar diets, effectively alleviated anxiety and depressive behaviors induced by CUMS. The underlying mechanism may be related to a reduction in pro-inflammatory cytokines and an increase in the expression of neurotrophic factors. Furthermore, the addition of BP alone to the diet did not produce the same protective effect as FP, indicating that the antidepressant effect of FP is not solely dependent on its main component, BP.

Our present study has several limitations. Although the CUMS model was employed to simulate a depressive state, inherent pathological and physiological differences between this animal model and human depression remain. Second, microbial composition was not analyzed, leaving a gap in understanding FP’s effects on the gut microbiota. Furthermore, while organ indices and weights were recorded, detailed monitoring of dynamic body weight changes was lacking, which may have influenced the assessment of overall metabolism and weight management. In addition, incorporating supplemental histological analyses, such as hematoxylin and eosin (HE) staining and immunohistochemistry, would have provided a more comprehensive visualization of FP’s protective effects on organs such as the brain and intestines. This approach would also have helped elucidate the associated tissue structural changes.

Future research should include metagenomics to explore the mechanisms of the gut–brain axis, as well as histopathological analyses to support the observed biochemical changes. A comparison of FP and known antidepressants is also necessary. Clinical trials in humans are needed to validate the antidepressant efficacy and safety of FP and to explore its potential as a complementary therapy for depression.

## Figures and Tables

**Table 1:** Record table of different groups of mice-fed diets.

Group	Feed	CUMS
Control Group(CG)	SD	N/A
SD	SD	Yes
SD + FP	50%SD + 50%FP	Yes
HFHSD	HFHSD	Yes
HFHSD + FP	50%HFHSD + 50%FP	Yes
HFHSD + BP	50%HFHSD + 50%BP	Yes

SD (standard diet), HFHSD (high-fat high-sugar diet), FP (formula powder), BP (buckwheat powder), CUMS (chronic unpredictable mild stress).

**Table 2:** Establishment schedule of chronic unpredictable mild stress.

Stressors	Implementation	Date of Implementation
Damp sawdust	Add 200 mL water to 100 g of bedding for 24 h.	Date 1, 10, 18, 25
Cage tilting	Tilt the cage at 45° and treat for 24 h.	Date 2, 8, 16, 22
Inversion light/dark cycle	Lights were not operated off at night, and rat cages were placed under a black cloth during the day (treated for 24 h).	Date 3, 11, 21, 26
Cage shaking	Shake each cage of mice horizontally at high speed for 5 min.	Date 4, 9, 15, 23
Forced swimming	Mice were placed in an open cylinder with water at 23 ± 1 °C and a depth of 20 cm for 6 min.	Date 5, 13, 19, 28
Restraint	Mice were confined to 50 mL centrifuge tubes with ventilation holes for 4 h.	Date 6, 12, 20, 27
Suspension	Mice were suspended 1 cm from the tail for 6 min.	Date 7, 14, 17, 24

**Table 3:** Real-time fluorescence quantitative PCR primer sequences.

Gene	Primer Sequence (F)	Primer Sequence (R)
GAPDH	TCTCCTGCGACTTCAACA	TGTAGCCGTATTCATTGTCA
TNF-α	ACTGAACTTCGGGGTGATCG	CCACTTGGTGGTTTGTGAGTG
IL-1β	CTTCAGGCAGGCAGTATC	CAGCAGGTTATCATCATCATC
IL-6	ACAAAGCCAGAGTCCTTCAGAG	AGGAGAGCATTGGAAATTGGG
BDNF	TCTACCCGACTCATGCTTGC	TCACTGTGAAGCCAGATCGC
TPH2	TCTACCCGACTCATGCTTGC	TCACTGTGAAGCCAGATCGC

The letters A, C, G, and T represent the four nucleotide bases of a DNA strand—adenine, cytosine, guanine, and thymine.

**Table 4:** Effect sizes (Cohen’s *d*) of FP in behavioral experiments.

Behavioral Experiment	Group	Cohen’s d
Immobility time on FST in mice	CUMS + SD vs. CUMS + SD + FP	1.29
	CUMS + HFHSD vs. CUMS + HFHSD + FP	1.58
Immobility time on TST in mice	CUMS + SD vs. CUMS + SD + FP	1.03
	CUMS + HFHSD vs. CUMS + HFHSD + FP	0.96
Distance of the central/total area on OFT	CUMS + SD vs. CUMS + SD + FP	−2.14
	CUMS + HFHSD vs. CUMS + HFHSD + FP	−0.18

## Data Availability

The original contributions presented in this study are included in the article. Further inquiries can be directed to the corresponding authors.

## Abbreviations

The following abbreviations are used in this manuscript:

SD	Standard diet
HFHSD	High-fat high-sugar diet
FP	Formula powder
BP	Buckwheat powder
CUMS	Chronic unpredictable mild stress
PCR	Polymerase Chain Reaction
FST	Forced swimming test
TST	Tail suspension test
OFT	Open field test
TNF-α	Tumor necrosis factor-α
IL-1β	Interleukin-1 beta
IL-6	Interleukin-6
BDNF	Brain-derived neurotrophic factor
TPH2	Tryptophan hydroxylase 2
